# Medicine Men of the World

**Published:** 1887-10-29

**Authors:** 


### Medicine Men of the World.

By the Man in the Crowd.

### II.—MEDICINE MEN IN SAVAGE LANDS.

To qualify oneself for the medical profession by undergoing excruciating physical torture after the manner described in a previous article, must be, one would think, a sufficiently unpleasant ordeal. There are, however, ceremonies which would strike most of us perhaps as even more dreadful. An American Vice-Consul describes an inaugural procedure, which he says came under his own observation among the Indians of the North. The candidate, he explains, had first been required to repair to the forest for a certain number of days, which were to be spent in strict solitude and unbroken fast. Starvation, by the way, nearly to the point of death, appears to be a very common preliminary to bodily torture in the case of candidates for the medical profession. Among civilised medicine-men there are not a few who would be glad to adopt the arrangement of the savage in this respect. With them the starvation comes later on in the career. It would be far more satisfactory to get it over at the outset, and have done with it once for all.

It has been conjectured by some that by reducing the vitality of the novitiate to the lowest possible point by fasting, his sensibility to pain is correspondingly lowered, and then the starving he undergoes assists him to endure the torture, and possibly expedites his recovery. It is said to be a fact that, terrible as the ordeal often is, it is very rarely attended by fatal or even serious results. But however this may be when swinging upon hooks or tearing the body by buffalo-thongs is the ordeal, a ten days' fast might not unreasonably be expected to prove a fatal preliminary to such a procedure as that recounted by this Vice-Consul. The famished wretch, brought in from the forest solitude, is led within the fences marking the limits of the "dog feast" which is to be his inaugural ceremony. A number of conjurers and braves of his tribe form a circle round him and perform a wild dance, in the midst of which a live dog—a white one, if possible—is brought to him by the presiding medicine-man. The candidate seizes the living sacrifice by the neck and a hind leg, and devours the animal alive. "The spectacle of this poor wretch, his face covered with blood, the howls and contortions of the suffering animal, and the yelling, dancing demons circling about in their monotonous dance, was appalling in the last degree." The dogs consumed at these revolting ceremonials are, it is added, generally of a small size, but in some instances large ones are given, and the neophyte at the conclusion of the business lies in a gorged and semi-conscious condition. Some who have been brought to the verge of starvation, and have afterwards taken a morsel of solid food too precipitately, have described the sensation experienced shortly afterwards as that of having a live lobster gnawing and pinching at the vitals. What a fearful time of it must be undergone by an unfortunate medical student who, gaunt and feeble from starvation, is required to devour a whole dog!

A man who has eaten a dog ought to be qualified for some, at least, of the operations he may be called upon to perform. Here, for instance, is what our traveller avows that he himself witnessed in the case of a young girl, one of the handsomest North American Indians he ever saw, who was troubled by some sort of ailment in her side. Cross-legged and naked, in the middle of the room, sat the medicine-man with a wooden dish of water before him, while twelve or fifteen other men sat round the lodge. The officiating practitioner had, when the traveller entered, apparently been engaging in some violent exercise, and was in a state of profuse perspiration. He sat down among the rest, therefore, apparently quite exhausted, and a younger medicine-man took his place in front of the bowl, and close beside the handsome young patient. Throwing off his blanket, he commenced singing and gesticulating as franctically as the elder medicine-man had apparently been doing, while the rest of the company raised a diabolical din with their tom-toms and other unmusical instruments.

Parenthetically it may be remarked that it is interesting to observe how very generally people in a primitive condition of civilisation appear to rely upon noise for the cure of bodily disease. It is, of course, to be explained by the fact that they believe in demoniacal possession as the cause of the malady; and not so many generations ago people here in England had a very similar method of putting the devil to the rout. The "passing bell" is indeed a relic of this sort of thing, with which we are all familiar to this day. As most people are probably aware, the tolling of the parish bell on the death of a parishioner is but a trifling modification of the old practice of tolling for the purpose of scaring off evil spirits during the passing away of the dying. A peal of bells was once held to be of sovereign efficacy for this purpose at all times; and we find Bishop Latimer arguing with the people of his day as earnestly on this point as one of our modern missionaries might have reasoned with these tom-tom men. "I tell you," said the bishop, "if the holy bells would serve against the devil, we would soon banish him out of all England; for I think if all the bells of England should be rung together at a certain hour there would be almost no place but some bells might be heard there, and so the devil should have no abiding place in England." It is, then, only eight or ten generations ago since our people here in England believed in beating their tom-toms in the church-steeples for spirit-scaring, and in this matter, at least, were therefore pretty much on a par with the Red Indians.

But to proceed with the cure of the young woman with a pain in her side. "After exercising himself for about half-an-hour, until the perspiration ran down his body, the young medicine-man dashed suddenly upon the patient, catching hold of her side with his teeth, and shaking her for a f minutes, while the young woman seemed to suffer great agony. He then relinquished his hold, and cried out he had got it, at the same time holding his hands to his mouth, after which he plunged them in the water, and pretended to hold down with great difficulty the disease which he had extracted." Very interesting it would have been to know what those twelve or fifteen quiet observers seated round the walls of the lodge were really thinking about as the cure proceeded. Had they, one wonders, any doubts about the efficacy of their own tom-toms and tambourines? Did they really believe the young medicine-man was grappling with the devil in the bowl of water there? Had they privately any cynical suggestions of a possible clue to the mystery of medicine when they thought of the doctor's fee? Who knows how closely they would sometimes correspond with their civilised fellow-creatures in these respects, could the truth have been known? At length, after great wrestling with the disease, lest it should spring out and return to its victim, he obtained complete mastery over it, and then turned round to the stranger in an exulting manner, and held up something between the finger and thumb of each hand, which had the appearance of a piece of cartilage, whereupon one of the Indians sharpened his knife and divided it in two, leaving one end in each hand. One of the pieces he threw into the water and the other into the fire, accompanying the action with a diabolical noise which only a medicine-man can make. After which, continues the narrator, he got up perfectly satisfied with himself, although the poor patient seemed anything but relieved by the violent treatment she had undergone.

(*To be continued.*)

